# NGF-mediated crosstalk: unraveling the influence of metabolic deregulation on the interplay between neural and pancreatic cancer cells and its impact on patient outcomes

**DOI:** 10.3389/fphar.2024.1499414

**Published:** 2024-12-11

**Authors:** Francesca Trentini, Virginia Agnetti, Martina Manini, Elisa Giovannetti, Ingrid Garajová

**Affiliations:** ^1^ Medical Oncology Unit, University Hospital of Parma, Parma, Italy; ^2^ Department of Medical Oncology, Lab of Medical Oncology, Cancer Center Amsterdam, Amsterdam UMC, VU University Medical Center (VUmc), Amsterdam, Netherlands; ^3^ Cancer Pharmacology Lab, AIRC Start-Up Unit, Pisa, Italy; ^4^ Cancer Pharmacology Lab, Fondazione Pisana per la Scienza, Cancer Pharmacology Iacome Department, San Giuliano Terme, Italy

**Keywords:** pancreatic cancer, NGF, neural invasion, metabolism, pain

## Abstract

Neural invasion is one of the most common routes of invasion in pancreatic cancer and it is responsible for the high rate of tumor recurrence after surgery and the pain generation associated with pancreatic cancer. Several molecules implicated in neural invasion are also responsible for pain onset including NGF belonging to the family of neutrophins. NGF released by cancer cells can sensitize sensory nerves which in turn results in severe pain. NGF receptors, TrkA and P75^NTR^, are expressed on both PDAC cells and nerves, strongly suggesting their role in neural invasion. The crosstalk between the nervous system and cancer cells has emerged as an important regulator of pancreatic cancer and its microenvironment. Nerve cells influence the pancreatic tumor microenvironment and these interactions are important for cancer metabolism reprogramming and tumor progression. In this review, we summarized the current knowledge on the interaction between nerves and pancreatic cancer cells and its impact on cancer metabolism.

## 1 Introduction

**FIGURE 1:**
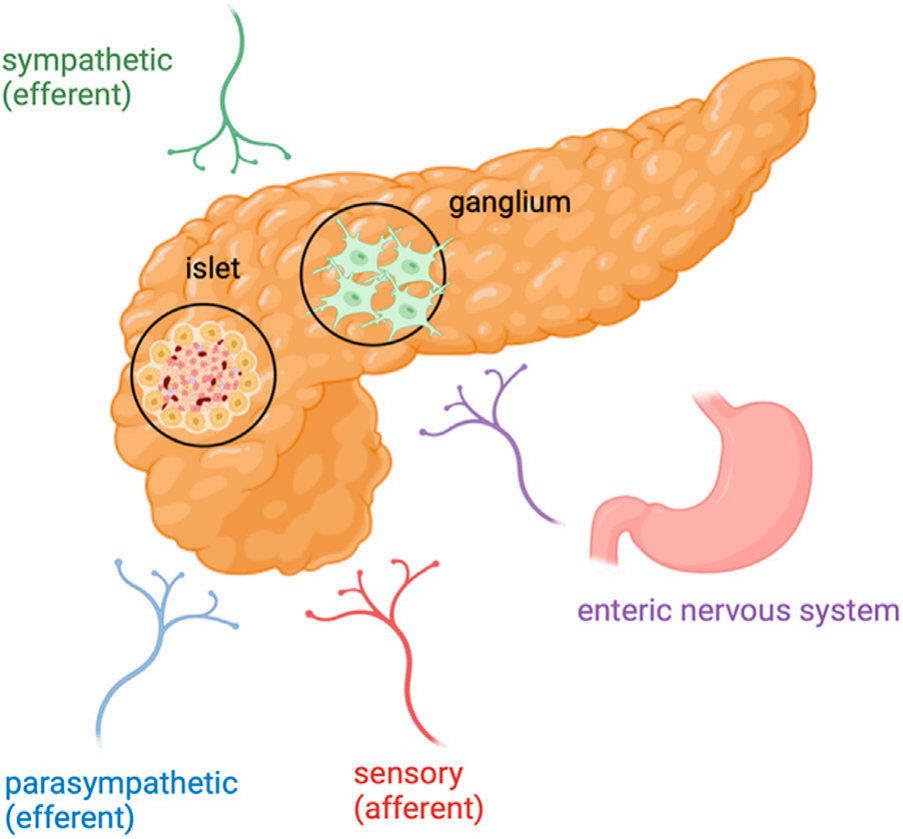
Pancreatic cancer and its innervation: In PDAC, nerve fibers include axons originating from the sympathetic and parasympathetic efferent nerve fibres, spinal and vagal afferent nerve fibres and also nerve fibres from the enteric nervous system. Created in BioRender. Garajová et al. (2024) https://BioRender.com/r60e612.

In Europe, pancreatic cancer is the fourth most lethal cancer in both genders. Its incidence is increasing and the prognosis of this type of cancer remains dismal over years (Conroy et al., 2023; Siegel et al., 2023; Digiacomo et al., 2021). The most common type of pancreatic cancer is pancreatic ductal adenocarcinoma (PDAC), which accounts for 80% of all pancreatic cancers. At the diagnosis, 30%–40% of patients suffer pain and 80% develop pain as cancer progresses (Moore and Adler, 2009). Importantly, pain in PDAC patients is associated with reduced performance status and decreased survival (Freelove and Walling, 2006). Both metabolic abnormalities and neural invasion (NI) are the hallmarks of PDAC. From metabolic point of view, PDAC can adapt to biosynthesis and energy intake through metabolic reprogramming in order to tolerate nutrient deficiency and hypoxic microenvironments. Regarding the NI, it has the highest prevalence in PDAC with a range varying between 70% and 98% (Liebl et al., 2014). The peripheral nervous system (PNS) can be viewed as a neuronal circuit that connects all body parts and organs to the central nervous system (CNS) and thus the brain in order to regulate muscle movements and sensations (Jobling et al., 2015). The pancreatic gland is innerved by both sympathetic and parasympathetic fibers of the autonomic nervous system, as well as from afferent sensory fibres (Figure 1). Importantly, peripheral nerves play a trophic function for epithelium, embryologically and into adulthood (Wang et al., 2024) and, therefore, play an important role in pancreatic tumorigenesis and present a potentional route of cancer spread (Selvaggi et al., 2022). The relationship between nerves and cancer cells are bidirectional. Cancer cells control nerves by induction of axonogenesis (the enlargement of nerves), neurogenesis (growth of neural progenitors) and neural reprogramming (the transforming of a sensory nerve into an adrenergic nerve) (Bakst et al., 2017). In this way, cancer cells supply the growth of new malignant epithelial cells. Moreover, NI is present even at the early stage of PDAC. In particular, the pancreas acinar cells has been found to migrate along sensory neurons in the spinal cord, proving the evidence that NI could be a potential mode of early cancer cell dissemination (De Oliveira et al., 2012).

**FIGURE 2:**
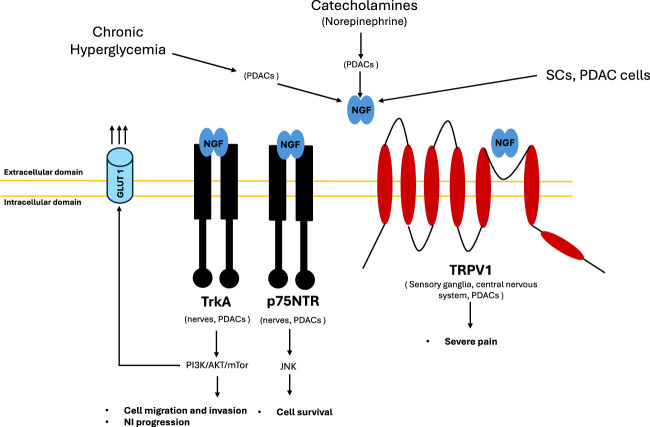
NGF interplay. NGF production is stimulated by different factors: metabolic (chronic hyperglycemia), neuronal (NE release) and directly by cells (SCs and PDAC cells). Chronic hyperglycaemia leads to axonal damage and demyelination and NGF overproduction by tumour cells. Hyperglycemia impair SCs proliferation and migration, as well as axon regeneration contributing to PDAC progression and aggravate the neural invasion process via nerve damage and inflammation. Catecholamines activate ADRB2 expressed on PDACs, resulting in NGF and BDNF overexpression, which in turn promotes neural invasion. NGF produced by PDACs binds TRKA receptors (with high affinity) and p75 NTR receptors (with low affinity) both expressed on PDACs and nerves establishing an autocrine and paracrine stimulation mechanism, respectively. TrkA receptor activates PI3K/AKT/mTOR pathway that mediates cell migration, invasion, neural invasion and GLUT1 upregulation favoring Warburg effect. p75NTR receptor activates JNK signaling that mediates cell survival. NGF produced by PDACs binds TRPV1 receptor causing severe pain. TRPV1 receptor is expressed on the sensory ganglia and in central nervous system, as well as by PDACs. Its stimulation leads to pain signals conduction and it induces the secretion of pain related neurotransmitters such as substance P and CGRP. Abbreviations: ADRB2, beta-2 adrenoceptor; CGRP, calcitonin gene related peptide; Glucose Transporter Type 1, GLUT 1; NGF, nerve growth factor; TrkA, tropomyosin receptor kinase A; p75NTR, p75 neurotrophin receptor; PDAC, pancreatic ductal adenocarcinoma; PI3K, phosphatidylinositol 3-kinases; JNK, c-jun N-terminal kinase; mTOR, mammalian target of rapamycine; NE, norepinephrine; TRPV1, transient receptor potential cation channel subfamily V member 1, SCs, Schwan cells.

Several molecules are involved both in NI and pain onset, including Nerve Growth Factor (NGF) which belongs to the family of neutrophins (Demir et al., 2016; Skaper, 2017; Ma et al., 2008; Kayahara et al., 2007). NGF is a molecule that is important for nutrition and promote the survival of sensory and sympathetic neurons *in vivo* and *in vitro*. NGF released by tumor cells can act on sensory nerves, especially on Transient Receptor Potential Vanilloid 1 (TRPV1) (Szallasi, 2024; Li L. et al., 2021; Bao et al., 2023) (Figure 2). Activation of this channel can lead to neurotransmitters’ release from peripheral nerve endings, including substance P (SP) and calcitonin gene-related peptide (CGRP) resulting in pain.

Therefore, we aimed to explore the connections between metabolism and NGF-correlated NI. There are several models to study NI in order to understand better the interactions between nerves, tumor cells, and the stroma. *In vitro* experimental models allow reasearchers to control the components and conditions of the cellular TME, these models have limitations, particularly in replicating the neural microenvironment and culturing peripheral nerves (Wang et al., 2024; Park et al., 2023). This might be overcome by *in vivo* experimental models which are able to recapitulate more truthfully the neuropathic changes that occur in human tumors, such as neural hypertrophy and neural remodeling. In particular, genetically engineered mouse models (GEMMs) are valuable tools to investigate NI, in particular the tumor-neural interactions at different stages of tumor progression (Le Magnen et al., 2016; Wang et al., 2021). For this narrative review, a comprehensive literature search was conducted on PubMed. We utilized various combinations of the following keywords and their synonyms: “pancreatic cancer,” “NGF”, “neural invasion,” “metabolism,” and “pain.” In our selection process, we considered abstracts, review articles and preclinical *in vivo* and *in vitro* experimental studies. The most relevant works were chosen based on their level of evidence, aiming to provide a vast overview of key findings and trends in this research area. Nowadays, clinical trials using neural invasion inhibition with or without drugs influencing PDAC metabolism and its impact on quality of life are lacking.

## 2 Neural invasion in pancreatic cancer and its clinical impact

NI can be defined as the existence of cancer cells along the sides of nerves and/or inside the epineural, perineurial and endoneural spaces of neuronal sheath including cases in which the cells encircle at least 33% of the nerves (Liebig et al., 2009). In epineural invasion, cancer cells directly touch the epineurium but do not penetrate it; in perineural invasion, cancer cells are within the perineural sheet; and, lastly, in endoneural invasion, cancer cells have invaded, through the perineurium, the endoneural sheet (Garajová et al., 2024). Besides intrapancreatic NI of cancer cells, neural alterations in pancreatic cancer are also represented by extrapancreatic NI. Interestingly, extrapancreatic nerve plexus invasion increases in parallel with the occurrence of intrapancreatic NI (Ceyhan et al., 2009). NI can be detected in PDAC, but also in pancreatic neuroendocrine tumours (pNET) as well as invasive intraductal papillary mucinous neoplasia (IPMN). In PDAC, the NI is typically more extensive, conversely, in pNET and IPMN is restricted to the perineurium only (Ceyhan et al., 2009).

NI is a complex situation: it is a consequence of an active interference between peripheral nerves and malignant cells (Liang et al., 2016). The dorsal root ganglion (DRG) is an essential part of the crosstalk between cancer cells and neurons (Saloman et al., 2016). DRG is an inflated nodule of the dorsal root of the spinal cord near the inner side of intervertebral foramen, which is responsible for receiving nerve impulses from receptors and transmitting them to the spinal cord, becoming the starting point of the pain conduction pathway. In the PDAC Tumor Microenvironment (TME), the DRG undergoes structural and functional reorganization. Structurally, it shows an increase of synapses. Functionally, it actively participates in the occurrence and development of NI through the expression and secretion of a variety of molecules: nerve growth factor (NGF), neurotrophins (NT), including glia cell line-derived neurotrophic factors (GDNF) and axon guiding molecules, chemokines, and adhesion molecules (Lian et al., 2020).

Abdominal or back pain is a characteristic feature in PDAC patients (Tieftrunk et al., 2015). To investigate whether neural changes were associated with abdominal pain, Ceyhan et al. (2009) recorded a pain history of patients and related it to the presence of intrapancreatic neuropathy. Enhanced neural density and hypertrophy were both strongly associated with the degree of NI and severe abdominal pain (Ceyhan et al., 2009). In clinical practice, extreme pain exacerbation was associated with retroperitoneal NI and have demonstrated the correlation with disease progression (Tieftrunk et al., 2015). In basic research, many molecules have been found to be involved in pain signalling associated with NI (Deborde et al., 2016). The invasion of tumor cells destroys the normal structure of the neural sheath and produces various molecules, contributing to neuropathic and inflammatory pain (Demir et al., 2015). The most common signalling pathway associated with cancer pain is the NGF pathway (Hirai et al., 2002). NGF, released by cancer cells, tumor-associated immune cells, and fibroblasts, stimulates the sensitive sensory nerves by interacting with transient receptor potential cation channel subfamily V member 1 (TRPV1) (Silverman et al., 2021). TRPV1, overexpressed in PDAC, acts on Na^+^ and Ca^2+^ channels to release pain related neurotransmitters, such as CGRP and substance P (Ceyhan et al., 2008a), resulting in severe pain (Hartel et al., 2006; Silverman et al., 2021). As mentioned earlier, also GDNF contributes to the NI process in PDAC patients. Surprisingly, a rich expression of GDNF was closely related to the degree of back pain before surgery or 12 months after surgical resection (Oseguera et al., 2007). Furthermore, GDNF secreted by tumor cells might upregulate TRPV1 expression, leading to more pain sensitivity (Malin et al., 2006). Moreover, ARTN is a protein that reduces the neuropathic pain and repairs neural impairment (Gardell et al., 2003). The increased expression of ARTN in PDAC cells and Schwann cells can also promote TRPV1 expression. Accordingly, the binding of ARTN to its receptor GFRa3 can enhance the functions of TRPV1 and the pain signalling pathway (Malin et al., 2006). Importantly, NGF and its high-affinity receptor *TrkA* are overexpressed in PDAC tissues compared with normal pancreas (Zhu et al., 1999). However, no difference in NGF and *TrkA* expression was observed between early (stages I and II) and advanced (stage III) stages and between well/moderately (grades 1 and 2) and poorly (grade 3) differentiated tumors. Nonetheless, tumors highly expressing NGF/*TrkA* exhibited increased incidence of NI and were associated with a higher degree of pain (Zhu et al., 1999). Similar results were obtained by Dang *et al.* confirming the association of high TrkA expression levels with more frequent NI, higher degree of pain and shorter overall survival (Hirai et al., 2002). In addition, TrkA has been significantly correlated with PNI in human tissue compared to tissue without PNI. NGF released by tumor cells promotes neuritic growth (D'Haese et al., 2013; Dai et al., 2007; Bapat et al., 2016; Ceyhan et al., 2008b), reduces apoptosis and increases proliferation of cancer cells leading to PDAC aggressiveness (Bapat et al., 2016; Bapat et al., 2011a; Miknyoczki et al., 1999; Miknyoczki et al., 2002; Zhu et al., 2002). Additional studies have reinforced the notion that overexpression of NGF and TrkA correlate with higher incidence of NI, increased pain and worse survival outcome in PDAC patients (Ceyhan et al., 2008b; Miknyoczki et al., 2002; Zhu et al., 2002; Larrieta et al., 2006; Zhu et al., 2001; Ma et al., 2008; Schneider et al., 2001). The association of upregulation of NGF and its TrkA receptor with lymph node metastases has been also documented (Kayahara et al., 2007). At mRNA level, the expression of TrkA and p75NTR were found to be associated with the presence of abdominal and/or back pain and NI (Demir et al., 2016; Dang et al., 2006). Specifically, tissue TrkA mRNA expression was higher among patients with more NI invasion, more severe pain and worse outcome. On contrast, high p75NTR mRNA expression were associated with a more favorable outcome (Demir et al., 2016; Dang et al., 2006).

NI can be considered as an independent risk factor for disease-free survival (DFS) and/or overall survival (OS) in PDAC patients (Hirai et al., 2002; Garajová et al., 2022). A retrospective study demonstrated that for patients with tumour cells infiltrated beyond the axon of the nerves, DFS and OS were 13.4 and 28.1 months, respectively. In patients whose disease did not reach the axon, DFS and OS were 32.9 months and 45.7 months, respectively (Wang et al., 2012). Not only DFS and OS but also the rate of peritoneal metastases has been shown to highly correlate with NI (Tanaka et al., 2019). Furthermore, several studies have shown that the postoperative survival rate of PDAC patients was negatively correlated with nerve infiltration (Mitsunaga et al., 2007). Being PNI-free is independently protective factor for long-term survival in PDAC patients (Stark et al., 2016). Moreover, the depth of nerve infiltration has also been shown to influence patient’s survival time (Hirai et al., 2002). These clinical studies support the oncogenic effects of NI in the initiation and development of PDAC (Zhang et al., 2024).

Glucose intolerance is often concurrently present in PDAC patients and confer worse prognosis (Meier and Giese, 2015). Interestingly, neuropathy is a well-known complication of diabetes and is associated with injury to the myelin sheet and neuroinflammation. It has also been hypothesized that hyperglycemia could promote NI in PDAC (Kumar et al., 2012; Sah et al, 2013). Indeed, PDAC patients with hyperglycemia showed a more elevated stage of NI compared to the patients with euglycemia. However, Sahin et al. and Li et al. demonstrated that diabetic patients had a significantly lower frequency of abdominal pain (Sahin et al., 2012; Li et al., 2015). It was hypothetized that hyperglycemia could exert a strong destructive effect on the fragile nerves, which promotes the progression from initial functional damage to late irreversible structural damage with or without attenuated pain (Li et al., 2011). Moreover, hyperglycemia supports the multiplication and invasion of pancreatic cancer cell lines and induces the expression of NGF in these cells, increasing the interaction between the nerves and cancer cells (Christianson et al., 2003). NGF regenerates the affected nerves by stimulating axon branching and may be a potential key player in the generation of pancreatic neuropathy in PDAC (Ceyhan et al., 2010). The overexpression of NGF can cause the hyperplasia of nerves, leading to an increase NI of pancreatic cancer cells. In turn, the expression of NGF can decrease the apoptosis of pancreatic cancer cells due to the collective signalling of NGF and TrkA in pancreatic cancer (Li and Ma, 2008). Moreover, hyperglycemia can impair Schwann cell proliferation and migration, as well as axon regeneration. These observations support the hypothesis that diabetes may contribute to PDAC progression and aggravate the NI process via nerve damage and inflammation. The consequence of destroyed nerves may exacerbate a poor prognosis either by enhanced cell proliferation and increased expression of NGF in pancreatic cancer cells and enhanced interactions of nerve and cancer cells, or by demyelinization and axonal degeneration of nerves, which facilitate cancer cells’ invasion of the nerves (Liang et al., 2016).

PDAC patients who received systemic chemotherapy before surgery have NI rate of 58% which was lower than that in those who did not receive neoadjuvant chemotherapy (80%) (Wang et al., 2012). Radiotherapy might also contribute to limiting NI, because it could remarkably reduce GDNF release in DRG, thereby inhibiting the invasion on cancer cells (Bakst et al., 2012). Sustained low-dose irradiation from iodine-125 seeds inhibited tumor growth and NI formation in preclinical models. Iodine-125 seeds planted in the local sites of tumors even relieved pain in PDAC patients (Lu et al., 2016). In conclusion, the identification of specific molecular targets or exploring new ways to inhibit the progression of NI and other neurological abnormalities may have a potential impact on PDAC pain treatment (Ceyhan et al., 2008a).

## 3 Perineural signaling pathways in pancreatic cancer

NI is a very complicated and complex process acting through a series of cellular interconnections mediated by secretory and contact molecules that activate signaling pathways (see also Table 1).

**TABLE 1:** Involvement of different molecules in neural invasion in pancreatic cancer.

Category	Molecules (upregulated >; downregulated <)	Production	Receptor	Role in NI	Refs.
Neurotrophines	NGF [>]	PDACs, SCs, fibroblast, immune cells	TrkA (high affinity)	Neural regeneration and repair	Skaper (2017), Shen et al. (2022), Campenot and MacInnis (2004), Sakamoto et al. (2010)
			TRPV1	Severe pain	
	BDNF [>]	PDACs	TrkB	Axonogenesis, EMT	Bakst et al. (2017)
	GDNF [>]	PDACs	GFRA1	Matrix degradation induction by metalloproteinases (cancer cell invasion), metastasis and pain	Shen et al. (2022)
	NT-3 [>]	PDACs	TrkB, p75NTR	Increased cancer invasiveness	Shen et al. (2022)
	NT-4 [>]	PDACs	TrKC, p75NTR	Increased cancer invasiveness	Shen et al. (2022)
	MDK [>]	PDACs	N-syndecan receptor 3	Pancreatic nerve homeostasis disruption	Petrusel et al. (2019)
	PTN [>]	PDACs	N-syndecan receptor 3	Pancreatic nerve homeostasis disruption	Petrusel et al. (2019)
Chemokines	CCL2 [>]	SCs	CCR2	Nerve damage	Bakst et al. (2017)
	CX3CL1 [>]	nerve cells	CX3CR1	PDACs migration	Marchesi et al. (2008)
	CXCL12 [>]	cancer-associated fibroblasts	CXCR4	PDACs migration and invasion	Marchesi et al. (2008)
	CCL5 [>]	PDACs	CCR5	Neural invasion	Zhang et al. (2020)
	CXCL10 [>]	peripheral sensory nerves	CXCR3	Neural invasion, nerve fiber hypertrophy and cancer pain	Renz et al. (2018), Gohrig et al. (2014)
	CCL21 [>]	peripheral sensory nerves	CCR7	Neural invasion, nerve fiber hypertrophy and cancer pain	Renz et al. (2018), Gohrig et al. (2014)
Neurotransmitters	Catecholamines [>]	sympathetic neurons	ADRB2	NGF and BDNF overexperssion	Jurcak et al. (2019)
Axon guidance factors	SEMA3D [>]	PDAC and nerve cells	PLXND1	Tumor cells migration along intrapancreatic nerves	Jurcak and Zheng (2019), Wang et al. (2015)
	SLIT2 [<]	PDACs	ROBO	Suppressor of metastasis and local tumor spread	Wang et al. (2015)
	Netrin-1 [>]	PDACs	Netrin-1 receptors	Neural invasion	Ridler (2016)

### 3.1 Neurotrophines

Neurotrophins normally play a role in the development, survival and function of neurons. NGF was the first to be identified in the neurotrophin family. Other neurotrophin family proteins include brain-derived neurotrophin factor (BDNF), glial-derivated neutrophic factor (GDNF), neurotrophin-3 (NT-3) and neurotrophin-4 (NT-4). The overexpression of neurotrophins in PDAC cells establishes a direct connection with nerves within the tumor microenvironment, contributing to tumor progression and invasion through neurotrophin-receptor interactions (Campenot and MacInnis, 2004). These interactions have been shown to be responsible for NI, tumor progression and pain (Campenot and MacInnis, 2004). Neurotrophins mediate their action by binding mainly to the three members tropomyosin related kinase (Trk) family of receptor tyrosine kinases (TrkA, TrkB, and TrkC) (Skaper, 2018). The binding of neurotrophins to their receptors triggers a retrograde signal that travels from the axon to the neuronal cell body and dendrites, promoting gene expression and axogenesis (Sakamoto et al., 2010). NGF is secreted by fibroblasts, immune cells, pancreatic cells and Schwann cells in response to neural injury to promote neural repair and regeneration (Garajová et al., 2024). NGF binds to the TrkA receptor activating the PI3K/Akt pathway, MAPK-ERK and PLCγ pathways (Petrusel et al., 2019). NGF levels were found to be elevated in PDAC tissue compared to non-tumoral pancreatic tissue and were linked to intense pain (Koulouris et al., 2017). NGF has two receptors: high-affinity TrkA and low affinity p75NTR receptor. In addition, NGF binds TRPV1 causing severe pain (Zhu et al., 2011). BDNF stimulates axonogenesis through TrkB, promoting NI through Epithelial-Mesenchymal Transition (EMT) (Silverman et al., 2021). NT-3 and NT-4 binds to TrkB and TrkC receptors, respectively, as well as to low affinity p75NTR receptor. GDNF binds to its receptor GDNF family receptor alpha-1 (GFRA1) and activates RET signal. The GDNF-GFRA1-RET signaling pathway promotes matrix degradation by inducing the production of metalloproteinases, thereby facilitating cancer cell invasion, metastasis, and pain (Campenot and MacInnis, 2004). Finally, two other neurotrophins involved in the mechanisms of NI are recognized: midkine (MDK) and pleiotrophin (PTN). By binding to the N-syndecan receptor 3, they disrupt pancreatic nerve homeostasis, creating a favorable environment for tumor cell invasion (Bakst et al., 2017).

### 3.2 Chemokines

Chemokines are peptide ligands acting as chemoattractant molecules. They are involved in tumor development, progression and NI. The chemokine CCL2, released by Schwann cells, binds to the CCR2 receptor on the surface of macrophages, triggering the production of cathepsin B. This enzyme breaks down collagen IV in the perineurium, leading to nerve damage (Marchesi et al., 2008). Nerves express on their surface and secrete CX3CL1, a ligand of CX3CR1 (Zhang et al., 2020). PDACs expressing CX3CR1 migrate to ligand-producing tissues, adhere to them, and initiate the process of NI (Zhang et al., 2020). Interestingly, the patients affected by PDAC with CX3CR1 overexpression are at a higher risk of locoregional recurrence (Zhang et al., 2020). Another chemokine signaling pathway, CXCL12/CXCR4, promotes the migration and invasion of PDAC cells by upregulating the expression of MMP-2 and urokinase-type plasminogen activator (uPA) (Xu et al., 2014). Hypoxia-inducible factor 1-alpha (HIF1-alpha), released from hypoxic tumor tissue, enhances the expression of VEGF, which subsequently upregulates CXCR4 on both tumor and endothelial cells, thereby promoting metastasis (Xu et al., 2014). C-C motif chemokine ligand 5 (CCL5)-CCR5 axis is another chemokine signaling pathway related to NI (Renz et al., 2018). Hirth *et al.* identified that chemokines, specifically CXCL10 and CCL21, can mediate cancer cell interaction, contributing to NI, nerve fiber hypertrophy and cancer pain through CXCR3 and CCR7 receptors expressed on PDAC cells (Hirth et al., 2020). In particular, they showed that neutralization of CXCL10 or CCL21 or their receptors in an orthotopic PDAC mouse model significantly reduced hypersensitivity and tumor-induced nerve hypertrophy without toxicity, discovering that chemokines might represent a possible target to prevent neural NI and to treat PDAC-associated pain (Hirth et al., 2020).

### 3.3 Neurotransmitters

The pancreas is an organ innervated by the autonomic nervous system, both by its sympathetic and parasympathetic components. A state of chronic stress has been shown to induce the release of high levels of circulating catecholamines (Gohrig et al., 2014). Catecholamines activate the beta-2 adrenoceptor (ADRB2), resulting in the overexpression of NGF and BDNF, which in turn promotes neural invasion (NI) (Gohrig et al., 2014).

### 3.4 Axon guidance factors

The axon guidance factors are molecules that guide the migration and positioning of neurons during brain development and act on endothelial cells by controlling angiogenesis (Jurcak et al., 2019). Semaphorin 3D (SEMA3D) and Plexin D1 (PLXND1) are axon guidance molecules that mediate the neuroplasticity mechanisms (Jurcak and Zheng, 2019). Annexin A2 (ANXA2) is a phospholipid binding protein that regulates exocytosis of SEMA3D (Jurcak and Zheng, 2019). SEMA3D is expressed by both tumor and nerve cells. Tumor cells expressing SEMA3D migrate along intrapancreatic nerves that display its receptor, PLXND1, thereby invading them (Jurcak and Zheng, 2019). SLIT2 is an axon guidance factor the binds its Roundabout receptor (ROBO) (Jurcak et al., 2019). SLIT2 acts as suppressor of metastasis and local tumor spread in experimental PDAC models (Jurcak et al., 2019). As SLIT2 in PDAC is downregulated, its re-expression inhibits PDACs chemotaxis to neural cells (Jurcak et al., 2019). Netrin-1, an axon guidance factor, has its expression upregulated through activation of the PI3K/AKT/NF-κB signaling pathway, triggered by the interaction between the membrane glycoprotein MUC4 and HER2, ultimately promoting increased neural invasion (NI) (Ridler, 2016). Mucin MUC4 is overexpressed in PDAC feeding this way (Ridler, 2016).

## 4 Nerve-cancer microenvironment in pancreatic cancer

**FIGURE 3:**
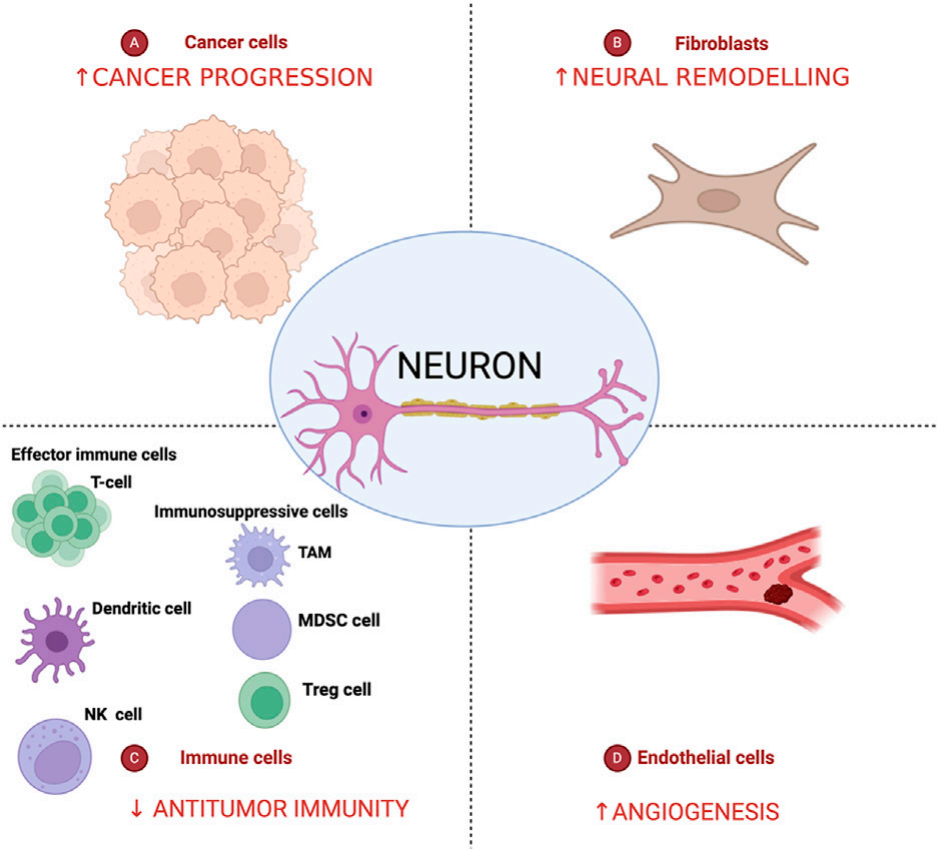
Nerves in the pancreatic cancer tumor microenvironment. Pancreatic cancer tumor microenvironment is characterized by an intricate crosstalk between cancer cells, cancer-associated fibroblasts, endothelial and immune cells and lead to decreasing of antitumor immunity and increase of tumor angiogenesis, neural remodeling, and cancer progression. Created in BioRender. Garajová et al. (2024) https://BioRender.com/c65d328.

Tumor Microenvironment (TME) is the complex of cellular and acellular structures that compose the tumor and communicate to each other orchestrating tumor development and progression (Wang et al., 2015) (Figure 3). During tumorigenesis, intrapancreatic nerves increase in size (neural hypertrophy) and number (increased neural density). These changes correlate with increasing pain severity and worsening prognosis in PDAC patients (Wang et al., 2015). NI it is a consequence of an active mutual interface between peripheral nerves and malignant cells, not just simple tumor cell penetration in the path where there is least resistance. Ligand-receptor binding interactions can activate specific signal transduction pathways that regulate growth factors, adhesion molecules, and matrix proteases, leading to the proliferation of cancer cells and the growth of nerve tissue (Liang et al., 2016). The DRG plays a crucial role in the communication between cancer cells and neurons, as it is responsible for receiving nerve signals from receptors and transmitting them to the spinal cord, serving as the initial point in the pain conduction pathway (Ridler, 2016). The relationship between tumor cells and nerve cells starts from the early stage of PDAC tumorigenesis (Zahalka and Frenette, 2020). Notably, tumor cells can reactivate signaling pathways that are involved in nervous system development and post-traumatic regeneration. This phase is mediated and fueled by the release of neurotrophins (Zahalka and Frenette, 2020).

Various histopathological and molecular alterations have been identified in PDAC specimens, including increased neural hypertrophy and density, perineural and endoneural invasion by cancer cells, changes in nociceptor expression, parenchymal immune cell infiltration, and the release of neurotrophic growth factors that are absent in the normal pancreas (Barreto and Saccone, 2012). Ceyhan et al. (2009) examined the presence of nerve hypertrophy and neural density in 149 surgical specimens of PDAC patients. In PDAC, nerve density and hypertrophy were increased by 14-fold and 2-fold, respectively, compared to non-cancerous tissue. PDAC patients who reported severe pain before surgery had a 3-fold greater nerve hypertrophy compared to those with mild pain or with no pain (Ceyhan et al., 2009). Finally, local inflammation induces the expression of TRPV1 in intrahepatic nerve endings, a channel that mediates sodium and calcium influx into neurons, promoting the generation of action potentials (di Mola and di Sebastiano, 2008). This releases SP and CGRP, two neurotransmitters which conduct pain signals from the parenchyma to the DRG (Barreto and Saccone, 2012). TRPV1 is overexpressed in PDAC tissue and is associated with the pain development (di Mola and di Sebastiano, 2008). This overexpression may also be responsible of hyperalgesia, hyperexcitability, and allodynia (Hartel et al., 2006).

Two-thirds of the TME of PDAC consists of an intense desmoplastic reaction, an invasion of stromal cells stimulated by the tumor to produce extracellular matrix (ECM) that occurs as a consequence of a chronic inflammatory state (Chu et al., 2007). Composition of stroma is tumor-type specific (Chu et al., 2007). It includes cellular and acellular components. Cellular components of the stroma include mesenchymal cells among which cancer-related fibroblasts (CAFs) and pancreatic stellate cells (PSC) (Chu et al., 2007). Perineurium originates from fibroblasts, and as a result, the change in fibroblasts in the TME can lead to changes in the permeability of perineurium and protection from injury, which directly affects the occurrence of NI (Chen et al., 2019). CAFs can release growth factors, cytokines, pro-angiogenic factors, and extracellular matrix proteins, which contribute to carcinogenesis, angiogenesis, and NI (Unterleuthner et al., 2020; Bunge et al., 1979; Kobayashi et al., 2019). CAFs also secrete Slit2, which stimulates Schwann cell proliferation and neural remodeling, resulting in increased nerve density (Secq et al., 2015). PDAC cells can trigger hedgehog signaling pathways in PSCs by releasing sonic hedgehog signaling molecules, facilitating cancer invasion and nerve dysfunction (Li et al., 2014). CAFs produce proteins related to prognosis and NI, for example, level of NECTIN1 is positively correlated with advanced tumor stage, NI and poor OS (Yamada et al., 2018).

A key component of the TME that orchestrates connections between PDAC, nerve cells and other TME cells is microglia. Schwann cells are peripheral nervous system support cells belonging to microglia part of endoneurium layer with function of myelin production (Bapat et al., 2011b). Normally, after a neuronal damage, Schwann cells are subjected to a reprogramming process by acquiring repair abilities (Deborde et al., 2016). Similarly, within tumor, Schwann cells dedifferentiate into an amyelin phenotype when subjected to biochemical signals and start to release neurotrophines like NGF, GDNF, BDNF stimulating neuronal growth (Deborde et al., 2016). Tumor hypoxic environment via HIF1-alpha induces immune cells and fibroblasts to release Granulocyte-Macrophage Colony Stimulating Factor (GM-CSF) promoting migration and proliferation of Schwann cells (Li J. et al., 2021). In turn, Schwann cells promote macrophage recruitment through the release of IL33 (Zhang et al., 2024) and CCL2 (Li J. et al., 2021). TAMs release cathepsin B which degrades collagen IV, a component of the perineurium, taking part in nerve injury process (Li J. et al., 2021). Thus, a mechanism of neuronal damage and repair feeds and maintains itself.

Moreover, a crosstalk between immune cells and nerve fibers exists (Heij et al., 2021). Immune cell clusters around the pancreatic nerves predominantly include mast cells, T-lymphocytes and macrophages (Demir et al., 2013). The type of immune infiltrate has a clear clinical impact. A retrospective study investigated the association between reported pain and the presence of specific types of immune cells in 20 tissue specimens from patients who underwent pancreatectomy for cancer (Demir et al., 2013). Patients who reported pain had pancreatic tissue infiltrated predominantly by mast cells, as opposed to those without pain where there was infiltration by T-lymphocytes. This observation, in addition to the fact that SP, CGRP and NGF bind to mast cells receptors and promote their degranulation and release of proteases, tryptases, supports a link between the immune and the nerve system in the generation on pancreatic cancer pain (Demir et al., 2013). Zwart *et al.* showed that patients with PDAC present heterogeneous areas in the immune-suppressive TME: the most immune-suppressive environment is in the central tumor area while less immune-suppressive side is further away toward the invasive part of the tumor and non-pathological tissue. In particular, the immune suppressive profile is composed of more macrophages, specifically M2, than T cells, inducing a pro-tumorigenic microenvironment contributing to development of metastasis and to chemoresistance (Zwart et al., 2023). Finally, the nerves generate neurotrophic factors and transmitters that attach to specific receptors and cause the migration of endothelial cells. This contribute to the process of angiogenesis (Jobling et al., 2015). Di Chiaro *et al.* introduced the concept of morphotypes, identifying three major morphological and functional variants (glandular, transitional and poorly differentiated) that can coexist in different proportions in PDAC cells, being associated with a different organization of the extracellular matrix: both *ex vivo* and *in vitro* evidences showed that the co-presence of different variants can induce resistance to anticancer therapy in PDAC or to the selection of treatment resistant cells, influencing NI (Di Chiaro et al., 2024). PDAC cells present a characteristic TME which is the cause of tumor cells growth and poor response to chemotherapy, showing a significant cancer-neuronal interaction (Ho et al., 2020). In keeping with these concepts, several preclinical studies have focused the attention on interrupting cancer-neuronal crosstalk, aiming to reduce cancer-associated pain and consequently to improve patients’ quality of life (Capodanno and Hirth, 2023).

## 5 Metabolic deregulation and its impact on neural invasion in PDAC

**FIGURE 4:**
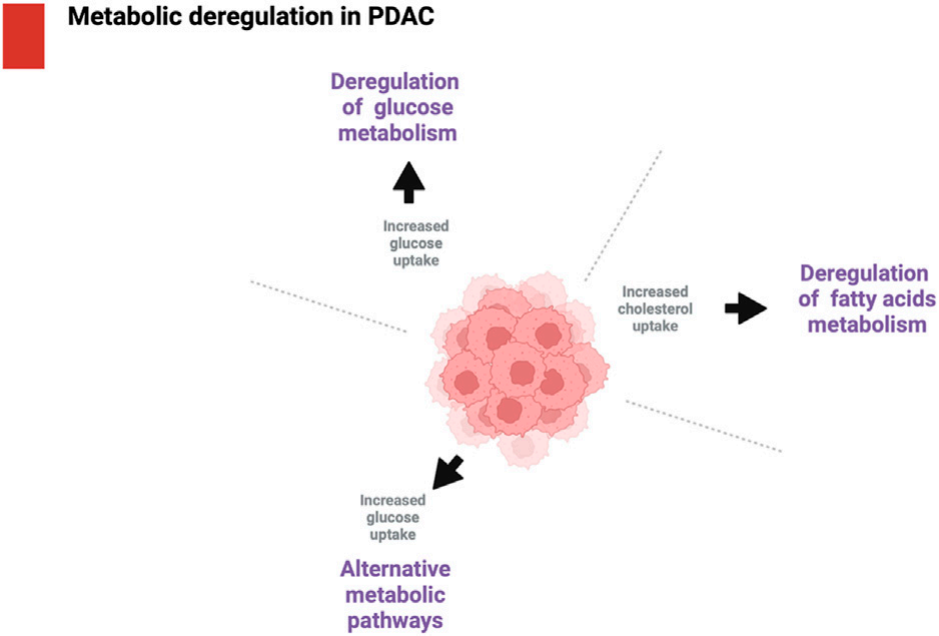
Metabolic deregulation in PDAC. The main metabolic deregulation in PDAC include deregulation of glucose metabolism, deregulation of fatty acids metabolism and alternative metabolic pathways such as autophagy. Created in BioRender. Garajová et al. (2024) https://BioRender.com/d53d820.

In the last years, there is an increasing attention to the alterations of cancer metabolism and the metabolic mediators have become the center of interest to identify possible “metabolic checkpoints” to block the cancer cell progression (Beloribi-Djefaflia et al., 2016). Bellow, we describe the principal metabolic alterations in PDAC, see also Figure 4.

### 5.1 Deregulation of glucose metabolism

Cancer cells use large quantity of glucose, which is fermented, even in the presence of oxygen, in lactate, as a result of the Warburg effect. Glycolysis is the main metabolic process that can produce adenosine triphosphate (ATP) and enhance anabolic activities. Glycolytic activity, that is regulated by glucose transporters and rate-limiting enzymes, plays a fundamental role in cancer metabolism (Hanahan and Weinberg, 2011)^.^ Altered glycolysis is considered as the major metabolic alteration in cancer (Yan et al., 2019). In PDAC, an enhanced glucose uptake thanks to the dysregulation of glucose transporters, comprehensive of facilitated transporters (GLUTs) and active transporters or symporters (SGLTs). In particular, GLUT-1 expression seems to be related to PDAC progression: a progressive increase of GLUT-1 is described from low-grade to higher-grade dysplastic lesions and it is strongly associated with KRAS mutation. On the other hand, it is not expressed in the acini or duct of normal pancreatic tissue (Yan et al., 2019). Kurahara *et al.* demonstrated that low expression of GLUT-1 in the primary PDAC tumor is associated with a better prognosis, increasing therapeutic response to neoadjuvant treatment in comparison to those patients with high GLUT-1 expression (Kurahara et al., 2018). In addition, Scafoglio *et al.* showed that SGLT2, an alternative glucose transporter, is overexpressed in PDAC (Scafoglio et al., 2015). This transporter can be the target of Canaglifozin, a SGLT2 inhibitor typically used to treat diabetes mellitus: in fact, as showed in PDAC xenograft models, Canaglifozin can reduce tumor growth and increase tumor sensitivity to PIK3 inhibitors (Hopkins et al., 2018). Approximately 90% of PDAC present KRAS mutations (Marchesi et al., 2008). The glycolytic activity in tumor cells depends on glycolytic genes whose expression is strongly associated with the hypoxic PDAC TME (Yun et al., 1979). In addition, KRAS can stimulate HIF1-alpha following PI3K pathway independently of hypoxia (Chen et al., 2001; Xu et al., 2014). The glycolytic activity is also related to a multi-step feedback regulation. In order to enhance glycolysis, cancer cells need to control intra-cellular PH levels by carrying lactate into the extracellular space via two major lactate transporters, monocarboxylate transporter 1 (MCT1) and monocarboxylate transporter 4 (MCT4), both strongly expressed in PDAC (Kong et al., 2016). The activity of MCT1 and MCT4 is induced by KRAS signaling (Baek et al., 2014). Another important mechanism that contributes to the deregulation of glucose metabolism is the increased glucose flux into anabolic pathways. The pentose phosphate pathway (PPP), consisting of both oxidative and non-oxidative phases, plays a crucial role in promoting cancer cell growth. It achieves this by generating ribonucleotides needed for DNA and RNA synthesis, as well as NADPH, a cofactor essential for detoxifying reactive oxygen species (ROS) and supporting macromolecule biosynthesis (Recktenwald et al., 2008). In particular, the oxidative arm is activated by KRAS signaling in order to enhance cancer cells proliferation (Weinberg et al., 2010). In the context of anabolic pathways, serine biosynthesis is important for the production of many molecules in different types of cancer (Possemato et al., 2011). In PDAC levels of serine are very low but, on the other side, KRAS signaling induces PDAC cells rely on *de novo* serine biosynthesis for survival, as it enables the production of nonessential amino acids such as glycine and cysteine. In fact, both glycine and cysteine serve as precursors to glutathione which is essential for the maintenance of ROS levels in the cell (Kamphorst et al., 2015).

Around 80% of patients with PDAC suffer from diabetes mellitus (DM) or glucose intolerance. Li *et al.* demonstrated that hyperglycemia is related with NI and can worsen prognosis of PDAC patients (Li et al., 2015). In patients affected by type 2 DM the risk of PDAC developing is 1.5 to 2-fold increased (Li, 2012). The hyperglycemia induces cells proliferation, promotes EMT and enhances the risk of metastasis (Jian et al., 2018). However, it has been recognized that PDAC can play a key role in the development of a form of new onset diabetes, called type 3c diabetes (pancreatogenic) which is characterized by high insulin levels and peripheral insulin resistance (Li J. et al., 2021).

### 5.2 Alteration of fatty acids metabolism

Lipid and fatty acids are part of the membrane matrix and helpful messengers. They are a fundamental source of energy, contributing to cancer progression (Chen et al., 2001; Beloribi-Djefaflia et al., 2016; Röhrig and Schulze, 2016). The lipidomic remodeling refers to the capacity of cancer cells to reorganize lipid metabolism with the aim of sustaining cellular proliferation and survival (Wang et al., 2015). Several studies have shown that alterations in lipid metabolism play an important role in cancer development and tumor progression across various cancer types (Broadfield et al., 2021). Notably, changes in cholesterol metabolism and transport are prevalent and significant in multiple cancers, including PDAC (Giacomini et al., 2021). PDAC has been shown to increase cholesterol uptake, enhance cholesterol synthesis, elevate storage of cholesteryl esters, reduce cholesterol efflux, all of which contribute to higher intracellular cholesterol levels (Guillaumond et al., 2015). Studies targeting cholesterol uptake, synthesis and esterification have generally shown suppressive effects on PDAC. Many changes in PDAC proteome and cell behavior induced by disrupted cholesterol flux were reverse by oleic acid, highlighting the critical role of fatty acids in maintaining cholesterol balance in PDAC, abolishing most effects caused by disturbance in cholesterol flux (Li et al., 2023) Walter et al. demonstrated that serum levels of fatty acid synthase (FAS) are elevated in patients with PDAC and IPMN (Walter et al., 2009). In PDAC, more than 62% of the reactions involved in *de novo* fatty acid synthesis, fatty acid elongation, and cholesterol biosynthesis pathways are upregulated, with an average increase of 1.5-fold. Key enzymes in this upregulation include citrate synthase, ATP citrate lyase, fatty acid synthase, and coenzyme A reductase (Islam et al., 2022). Higher expression of FAS is associated with shorter survival and with a reduced response to gemcitabine therapy in PDAC (Tadros et al., 2017). Furthermore, the exogenous absorption of fatty acids is closely linked to the expression of the CD36 transporter, which can also influence gemcitabine resistance in PDAC by modulating anti-apoptotic proteins (Kubo et al., 2020). For this reason, in the future, CD36 can represent a possible therapeutic (Kubo and Eguchi, 2020). Ding *et al.* demonstrated that an appropriate nutritional treatment can inhibit the kinases responsible for driving the neoplastic process. In fact, an omega 3-enriched diet can help to decrease lesion penetrance through reduced levels of phosphorylated AKT/protein kinase B (pAKT), while, on the other hand, an omega 6-enriched diet accelerated tumor formation (Ding et al., 2018). Mohammed et al. studied the role of omega-3 PUFAs on PanINs (pancreatic intraepithelial neoplasia) and consequent progression to PDAC using omega-3 fatty acid desaturase (Fat-1) in transgenic mice: the study hypothesized that an omega-3 based diet might be useful for PDAC chemoprevention (Hidaka et al., 2015; Chen et al., 2019).

### 5.3 Alternative metabolic pathways

PDAC cells can utilize various nutrient sources to meet their energy needs. For instance, they engage in self-digestion (autophagy) and absorb external nutrients via macropinocytosis, driven by lysosome activation (Perera et al., 2015). These different sources, like sugars, amino acids, nucleosides, and fatty acids are generated from these nutrient salvage pathways to induce tumor progression (Onodera and Ohsumi, 2005). Autophagy, a lysosome-mediated self-digestion process, is fundamental for cancer cells since it contributes to maintain cells activity under stress conditions such as nutrient deprivation and hypoxia (Boya et al., 2013). In PDAC autophagy helps to maintain energy homeostasis by removing reactive oxygen species (ROS), though it can also reduce DNA damage to enhance tumor cell proliferation (Yang and Kimmelman, 2011). For these reasons, the inhibition of pancreatic cells autophagy can represent a possible key to block the adaptive metabolic mechanisms in PDAC (Yang et al., 2011). For example, the use of combining chemotherapy with hydroxy-chloroquine in patients with PDAC has shown an improved response to therapy (Zeh et al., 2020). In keeping with this, other studies highlighted the role of combined inhibition of ERK/MAPK pathway and autophagy, leading to several clinical trials about ERK/MAPK pathway inhibitors in association with hydroxy-chloroquine (NCT04145297; NCT03825289; NCT04132505) (Piffoux et al., 2021). In addition to this, there is a specific type of autophagy, called ferritinophagy, which is fundamental to maintain iron homeostasis in PDAC cells (Mancias et al., 2014). In fact, the loss of autophagy is related to mitochondrial dysfunction due to abrogation of succinate dehydrogenase complex iron sulfur subunit B (SDHB) expression (Mukhopadhyay et al., 2024). Mukhopadhyay *et al.* demonstrated that cancer-associated fibroblasts can provide bioavailable iron to cancer cells, provoking resistance to autophagy ablation, and that a low-iron diet can improve a response to autophagy inhibition therapy in PDAC-bearing mice (Mukhopadhyay et al., 2023) Macropinocytosis can help PDAC cells to acquire nutrients by taking up the extracellular macromolecules. As mentioned before, in PDAC, KRAS mutations present a critical role in reprogramming metabolism (Ying et al., 2012). In particular, the SDC1 (syndecan-1) protein is activated by KRAS on the cell surface of PDAC cells and it can induce micropinocytosis representing a potential diagnostic and treatment target in the future (Yamada et al., 2018; Yao et al., 2019).

## 6 Conclusion

Pancreatic cancer is an aggressive disease often accompanied by wasting condition including intractable pain. The crosstalk between the nervous system and cancer cells has emerged as an important regulator of tumor microenvironment, as well as metabolic reprogramming in PDAC. For these reasons, therapies able to inhibit nerve invasion strengthened by pancreatic cancer cells might have a strong rationale to contrast this dismal disease. Indeed, the possibility of antalgic ancillary therapies in addition to chemotherapy could improve patients’ quality of life. Pain is one of the most disabling symptoms, negatively impacting patients’ quality of life, with adverse psychological effects that influence their perception of the disease, general functional abilities, and capacity to endure the therapy itself. Translating scientific expertise into practices that improve patient care would enable a more holistic approach to patient and disease management.
